# Notes on Health Resorts

**Published:** 1899-12-16

**Authors:** Robert Saundby


### THE INSTITUTIONAL WORKSHOP.

### NOTES ON HEALTH RESORTS.

### MARTIGNY, CONTREXEVILLE, AND VITTEL,

By Robert Saundby, M.D., LL.D., F.R.C.P.

(*Continued from page* 169.)

As has been already noted Contrexéville is not a place which has many natural advantages as a health resort. It is relatively low, shut in, close, rather damp, without the attraction of a pretty neighbourhood, so that it is not reasonable to explain the benefit which patients derive by simple rest and pleasant surroundings. But I may add that the patients who were good enough to give me their experience had, as a rule, tried other places before coming to Contrexéville.

If we look at the analysis of the Pavillon Spring, we see that it contains only 2·384 per 1,000 of mineral matter, of which the chief constituents are sulphate and carbonate of lime, these together yielding 1·967 parts or four-fifths of the whole of the inorganic matter. It is probable that it is to the bicarbonate of lime that the water owes its therapeutic action. If we compare the springs at the three places, it is only in the quantity of this salt that any essential difference can be found :—

Comparison of the Analyses of the Mineral Waters of Martigny, Vittel, and Contrexeville.

| | | Martigny (Source No. 1). (Analysed by Professor Jacquemin.) | Vittel (Grande Sce). (Analysed by M. Ossian Henry.) | Contrexéville (Sce du Pavillon). (Analysed by M. Debray.) |
|---|---|---|---|---|
| Free carbonic acid gas | | traces | 1/10 by volume | 0·0800 |
| Bicarbonates | of lime | 0·1620 | 0·1850 | 0·4020 |
| | of magnesium | 0·1750 | 0·0790 | 0·0350 |
| | of soda | 0·0160 | — | — |
| | of iron | 0·0090 | 0·0100 | 0·0040 |
| | of lithia | 0·0320 | — | 0·0070 |
| Sulphates | of lime | 1·4240 | 0·4400 | 1·5650 |
| | of soda | 0·2290 | 0·3260 | 0·2360 |
| | of magnesium | 0·3300 | 0·4320 | 0·0300 |
| | of strontium | — | traces | — |
| Silica | | — | — | 0·0150 |
| Silicates | of soda | 0·0532 | 0·0470 | — |
| | of lime | 0·0029 | | — |
| Phosphate of lime | | 0·0028 | — | — |
| Chloride | of sodium | 0·0950 | 0·2200 | 0·0040 |
| | of magnesium | — | | — |
| | of potassium | 0·0120 | — | 0·0060 |
| | of calcium | traces | — | — |
| Arsenic | | traces | traces | traces |
| Iodine | | — | traces | — |

Taking the bicarbonate of lime alone we find the following proportions: Per mille—Martigny, 0·1620; Vittel, 0·1850; Contrexéville, 0·4020. Their mutual relations may be expressed thus: Martigny, 40; Vittel, 46; Contrexéville, 100. That is to say, we require ten glasses of Martigny Source No. 1, or nearly nine glasses of Vittel Grande Source, to obtain the same dose as is contained in four glasses of Contrexéville (Pavillon) water. Besides the obvious inconvenience of these large quantities they involve a disproportionate dose of other constituents, notably of the aperient sulphates of soda and magnesia, which are present in the three springs in the following quantities: Martigny, 0·5590; Vittel, 0·7580; Contrexéville, 0·2660. So that besides having to drink from two to two and a half times as much water patients at Martigny and Vittel would get respectively twice and thrice the dose of aperient sulphates. It may also be observed that the Pavillon spring contains the least iron of any, which is a great advantage inasmuch as many gouty persons are very intolerant of this metal.

The Pavillon spring is very remarkable for the quantity of its water, which is poured out at the rate of 200,000 litres in 24 hours. This is far in excess of the needs of drinkers, or even of bottling for export, which is constantly going on, the abundant overflow supplying the douches and baths. We must reckon this splendid water supply as not the least among the advantages of Contrexéville, where neither pumping nor storage is needed. If, moreover, we recall the points already enumerated, the relatively small dose, the mildness of its aperient action, and the minimum amount of iron present, we can understand that Contrexéville is favoured by Nature beyond compare in the possession of this noble well.

The arrangements for baths and douches are excellent, all the modern improvements having been introduced. The general and local douches are, perhaps, the most usually prescribed, and the former are as has been hinted, particularly useful and agreeable by their bracing effect upon persons who are living in a somewhat relaxing place and undergoing a trying course of treatment. The water-closets and urinals at the Etablissement are old-fashioned, and need reconstruction.

The names of the doctors practising at Contrexéville are given in the following alphabetically-arranged list: Drs. Aymé, Boichox, A. Boursier, Debout-d'Estrées, Graux, Mabboux, and Thiéry. The season is the same as at Martigny, and is given officially as from May 20th to September 20th.

I cannot conclude this notice without expressing my sense of the excellent administration of the Etablissement, casino, and hotel, and of the immense pains which are taken to provide for the comfort and enjoyment of visitors.

Vittel.

The last of the three stations is Vittel, only three miles from Contrexéville, and consequently quite within walking distance. The park and Etablissement of Vittel are situated about three-quarters of a mile from the village of the same name, and are admirably placed in a wide open valley surrounded by pretty rising hills, on the slopes of which are the Casino, the hotels, and a few villas. The spacious park is well laid out, planted with fine trees arranged with elegance and taste, and brightened with borders of flowers which grow here luxuriantly. There are many fine and handsome hotels in and near the park, the largest being the Hotel de l'Etablissement, which stands between the Casino and the Etablissement, communicating with each by covered ways. The public buildings are all new, handsome, and well adapted for their purpose. The sanitary arrangements are specially noteworthy, being models of what they should be, and excellently well kept. Visitors may reserve w.cs. for their own use by the payment of a moderate fee. I can give nothing but praise to all these matters ; no expense has been spared, everything has been done well, and the result is most satisfactory. In the park tennis, croquet, and (clay) pigeon-shooting may be enjoyed every afternoon; there are a gymnasium, a bicycle school with a very good track, and a fencing school; while the band plays twice a day, every morning and afternoon. Finally, there are no bad smells, and neither damp nor night mists are complained of. In the Casino there is a theatre, which at my visit was supplied with a moderately good company of actors. There was very little gambling, even Petits Chevaux being usually played for 50 centime stakes. The reading-room was well supplied with papers but was bare looking, and the service of refreshments was not very good. In all these respects Vittel might be smartened up with advantage. During the past season a coach has been running every afternoon, starting from the park and visiting the various places of more or less interest within 20 miles radius, including Domrémy, the home of Jeanne d'Arc.

The lighting is by acetylene gas, which gives most brilliant illumination, and for outside purposes seems excellent. Indoors complaints were heard that it gave rise to a garlic-like smell which was undoubtedly present, although on the continent one does not usually look to acetylene gas for the source of this unpleasant odour.

The following is a list of the physicians practising at Vittel, arranged in alphabetical order: Dr. Bouloumié, Dr. Galland-Gleize, Dr. Patézon, and Dr. de Tymowsky. The season is the same as at Contrexéville, that is, from the middle of May until the third week in September. Vittel has four wells: (1) The Grande Source (diuretic); (2) the Source Salée (laxative); (3) the Source Marie (diuretic and laxative); (4) the Source des Demoiselles (chalybeate). The first three are in the Etablissement and the fourth is in the park, at only a very short distance, but it will soon be brought under the same roof as the others.

ANALYSIS OF VITTEL WATERS.

| | Grande Source. | Source Salée. | Demoiselles. |
|---|---|---|---|
| Free carbonic acid | 1/10 volume | 0·2836 | 0·080 |
| Bicarbonate of lime | 0·185 | 0·3188 | 0·730 |
| magnesia | 0·079 | 0·0028 | |
| soda | | — | — |
| iron | — | 0·0005 | 0·041 |
| (with manganese) | 0·010 | — | — |
| Sulphate (anhydrous) of lime | 0·440 | 1·4215 | 0·440 |
| magnesia | 0·432 | 0·8216 | 0·610 |
| soda | 0·326 | — | |
| strontium | traces | — | — |
| lithium | — | traces | — |
| Chloride of sodium | 0·220 | 0·0155 | — |
| magnesia | | — | — |
| Silica, alumina, calcium-phosphate | 0·047 | — | 0·480 |
| Potash and ammonia | | — | |
| Iodine. Arsenic | | — | |
| Organic matter | | 0·0118 | — |
| Aluminium | — | 0·0014 | — |
| Silicate of sodium | — | 0·0342 | — |
| ,, ,, potassium | — | 0·0118 | — |
| Total | 1·739 | 2·9226 | 2·381 |

The above table shows that Vittel water possesses the same characters as that of Contrexéville, but is weaker in diuretic salts. The Grande Source, which is that chiefly used, is considerably weaker in every respect but it is exceedingly pleasant water, well tolerated in large quantities, and appears to suit some cases or persons even better than the waters of Contrexéville. The Source Salée contains so much sulphate of magnesia that it must be and is regarded as a purgative water. The Source Marie is intermediate between these two, and is prescribed to patients as a stage after taking the Source Salée and previous to commencing the Grande Source. As it is not for the treatment of anæmia that we send our patients to Vittel we are not much concerned with the Source des Demoiselles, which is, however, the strongest chalybeate well which has fallen under my notice in the district.

In spite of the admirable bathing arrangements, baths and douches are seldom prescribed at Vittel. It is probable that this is due to difficulty in the water supply, all the water having to be pumped into a water-tower in order to keep up a continuous stream. This is a natural disadvantage which cannot be easily exaggerated; and, in my opinion, it will be found to be the insuperable obstacle to the development of Vittel into a formidable rival to Contrexéville.

The Source Salée is that which is prescribed for liver troubles, biliary lithiasis, &c.; while the Grande Source is used for those urinary diseases, renal calculus, glycosuria, gravel, gouty cystitis, &c., in which the waters of the Vosges seem to have a specific action.

